# Empagliflozin Inhibits Hepatic Gluconeogenesis and Increases Glycogen Synthesis by AMPK/CREB/GSK3β Signalling Pathway

**DOI:** 10.3389/fphys.2022.817542

**Published:** 2022-03-01

**Authors:** Xiaochen Yu, Ziyu Meng, Ting Fang, Xiaohuan Liu, Ying Cheng, Linxin Xu, Xiangyang Liu, Xiaoyu Li, Mei Xue, Ting Li, Bei Sun, Liming Chen

**Affiliations:** NHC Key Laboratory of Hormones and Development, Tianjin Key Laboratory of Metabolic Diseases, Chu Hsien-I Memorial Hospital & Tianjin Institute of Endocrinology, Tianjin Medical University, Tianjin, China

**Keywords:** SGLT2 inhibitor, empagliflozin, gluconeogenesis, glycogenesis, AMPK/CREB/GSK3β signalling pathway

## Abstract

Increases in glucose production and decreases in hepatic glycogen storage induce glucose metabolic abnormalities in type 2 diabetes (T2DM). Empagliflozin, a sodium-dependent glucose transporter 2 (SGLT2) inhibitor, is an effective hypoglycemic drug; however, the effects of empagliflozin on hepatic gluconeogenesis and glycogenesis are still unclear. In this study, we investigated the effects and mechanisms of empagliflozin on hepatic gluconeogenesis and glycogenesis *in vivo* and *in vitro*. Empagliflozin was administered *via* gavage to db/db mice for 8 weeks, and human hepatocyte HL7702 cells were treated with empagliflozin after palmitic acid (PA) stimulation. Compared with the control db/db mice, empagliflozin-treated mice showed a significant reduction in urine glucose levels, blood glucose levels, body weight and intraperitoneal glucose tolerance test (IPGTT) blood glucose levels. Moreover, the expression levels and activities of key gluconeogenesis enzymes PEPCK and G6Pase were dramatically reduced in the empagliflozin-treated mice, and the protein expression levels of AMPK/CREB/GSK3β signalling pathway-related molecules were significantly changed. In HL7702 cells, empagliflozin ameliorated glucose production and PEPCK and G6Pase expression and activity. Empagliflozin could also prevent the decreases in glycogen content and regulate the protein expression levels of AMPK/CREB/GSK3β signalling pathway-related molecules. Then, we selected the AMPK agonist AICAR and inhibitor compound C to further verify the effects of the AMPK signalling pathway on hepatic gluconeogenesis and glycogen synthesis. The results of the 5-Aminoimidazole-4-carboxamide1-β-D-ribofuranoside (AIACR) intervention in HL7702 cells were consistent with those of empagliflozin treatment, and the effects of empagliflozin were abolished by compound C. In summary, empagliflozin could maintain glucose homoeostasis by reducing gluconeogenesis and increasing glycogenesis through the AMPK/CREB/GSK3β signalling pathway.

## Introduction

In recent years, the number of T2DM patients has increased rapidly worldwide. One of the major pathophysiological changes in T2DM is active endogenous glucose production (Kahn et al., 2014). Previous studies have indicated that insulin resistance influences insulin signal transduction and inhibits hepatic gluconeogenesis and glucose production (Schenk et al., 2008). Because of reduced insulin secretion and insulin resistance, hepatic glycogen synthesis is reduced in T2DM (Velho et al., 1996).

The liver plays an important role in maintaining the homeostasis of glucose uptake, decomposition, and storage as well as gluconeogenesis and glycogenolysis (Park et al., 2018). Hepatic glucose production accounts for approximately 90% of endogenous glucose production (Moore et al., 2012). After overnight fasting, most of the glucose released into the circulation originates from hepatic gluconeogenesis while approximately 20% of the glucose originates from renal gluconeogenesis (Alsahli and Gerich, 2017). Gluconeogenesis is mainly regulated by phosphoenolpyruvate carboxykinase (PEPCK) and glucose-6-phosphatase (G6Pase), which are both key enzymes in the process of gluconeogenesis. PEPCK and G6Pase are upregulated by glucagon during fasting but downregulated by insulin during hyperglycaemia (Wang et al., 2014).

AMP-activated protein kinase (AMPK) is an indispensable cellular energy sensor that is widely involved in the regulation of various physiological processes, including glucose and lipid metabolism, mitochondrial homeostasis and autophagy (Herzig and Shaw, 2018). AMPK is activated by phosphorylation of Thr172 in the alpha subunit, which then improves glucose homeostasis by inhibiting gluconeogenesis-related genes (Kim et al., 2008; Agius et al., 2020). Previous studies have demonstrated that phosphorylated AMPK induces an increase in the phosphorylation level of CREB-regulated transcription coactivator 2 (TORC2), which inhibits the activity of cAMP-responsive element binding protein (CREB) to decrease hepatic gluconeogenesis (Koo et al., 2005). Glycogen synthase kinase-3β (GSK3β) and glycogen synthase (GS) regulate glycogen synthesis (Basu et al., 2005). GS is a key enzyme for regulating the final step of glycogen synthesis, whose activity is regulated by GSK3β (Liu et al., 2015). Increased AMPK phosphorylation levels induce an increase in the phosphorylation level of GSK3β, which results in the inactivation of GSK3β and a higher expression level of GS and ultimately increases glycogen synthesis (Horike et al., 2008).

Sodium-dependent glucose transporter 2 inhibitors, including canagliflozin, dapagliflozin and empagliflozin, are effective hypoglycemic drugs. SGLT2 is a transporter with low affinity and high capacity, and it is mainly expressed in the renal proximal convoluted tubule S1 segment and responsible for reabsorbing glucose from renal tubular filtration fluid (Han et al., 2008). Previous studies have reported that the hypoglycaemic effect of SGLT2 inhibitors is independent of insulin, and they mainly depend on the reduction of renal glucose reabsorption to increase glycosuria reduction (Heerspink et al., 2018). In addition, SGLT2 inhibitors can also promote body weight loss and cardiovascular benefits (Xu et al., 2017; Li et al., 2019; Xue et al., 2019).

Recent studies have demonstrated that SGLT2 inhibitors can increase the phosphorylation level of AMPK *in vivo* (Zhou et al., 2018; Chiang et al., 2020). AMPK is activated through phosphorylation of Thr172 in the alpha subunit to maintain glucose homeostasis (Agius et al., 2020). However, the exact effects and molecular mechanism of SGLT2 inhibitors on hepatic gluconeogenesis are still unknown. In this study, we investigated the effects and molecular mechanism of empagliflozin on hepatic gluconeogenesis and glycogen synthesis *in vivo* and *in vitro* to more comprehensively demonstrate the pharmacological effects of empagliflozin.

## Materials and Methods

### Animal Experiments

The db/db mice was the BKS background. In the db/db mice, the leptin receptor was mutated to disrupt the leptin signalling pathway, which resulted in significant obesity and higher fasting blood glucose levels at 6 weeks with increased water intake and urine volume. When the mice were 8–12 weeks old, the above symptoms were the most obvious, which was also accompanied by diabetic nephropathy and other complications. As the control group, db/m mice were heterozygous mice in the same background without metabolic disorder symptoms. Six-week-old db/m and db/db male mice were obtained from the Model Animal Research Center of Nanjing University. *Ad libitum* access to water and chow was provided, and the mice were housed at a constant room temperature of 20 ± 2°C under a 12 h light/dark cycle in the Experimental Animal Center of Tianjin Medical University, Chu Hsien-I Memorial Hospital. After adjusting to the environment for 2 weeks, twelve db/db mice were randomly divided into two groups (*n* = 6 mice/group) and fed a normal diet. According to the equivalent dose conversion formula of pharmacological experimental animal body surface area, we calculated the equivalent doses for humans and mice. One group of db/db mice was gavaged with 3.8 mg/kg/d empagliflozin for 8 weeks, and the other group of mice was gavaged with the same volume of distilled water. As a control group, db/m mice were fed a normal diet. Urine glucose levels, blood glucose levels and body weights were measured every 2 weeks. IPGTT was performed in each group of mice after empagliflozin treatment. Blood samples from the mouse tail vein were measured by a glucose analyser (Roche ACCU-CHEK, Basel, Switzerland). Urine glucose levels were measured by electrode method with a routine urine analyser at the Department of Clinical Laboratory of Tianjin Medical University, Chu Hsien-I Memorial Hospital (Roche Cobas U411, Basel, Switzerland). After empagliflozin treatment for 8 weeks, the mice were sacrificed under anaesthesia with 5% isoflurane inhalation. Liver tissues and blood samples were collected for further study. Our animal experiments were approved by the Experimental Animals Ethics Committee of Tianjin Medical University, Chu Hsien-I Memorial Hospital and Tianjin Institute of Endocrinology.

### Cell Culture and Drug Treatment

The human liver normal cell line HL7702 was purchased from ATCC (Manassas, VA, United States). HL7702 cells were cultured in RPMI 1640 medium (Gibco, New York, NY, United States) supplemented with 10% FBS (ExCell Bio, Shanghai, China), 500 μL penicillin 10,000 U/mL and streptomycin 10,000 μU/mL in cell culture incubators at 37°C, 95% air and 5% CO_2_. In some experiments, HL7702 cells were treated with 8 μmol/L empagliflozin for 24 h. HL7702 cells were also treated with 0.75 mmol/L PA (Sigma-Aldrich, P9767, St. Louis, MO, United States) for 24 h (Gao et al., 2010; Fang et al., 2019). Cell viabilities were detected using a cell counting kit-8 (CCK8, Vazyme, A311-01, Nanjing, China) assay at 450 nm according to the instructions.

In the 5-Aminoimidazole-4-carboxamide1-b-D-ribofuranoside (AIACR) treatment experiment, HL7702 cells were divided into four groups: normal control group, PA intervention group, PA combination with empagliflozin intervention group, and PA combination with 10 μmol/L AIACR (MCE, HY-13417, Trenton, NJ, United States) intervention group. In the Compound C treatment experiment, HL7702 cells were divided into four groups: normal control group, PA intervention group, PA combination with empagliflozin intervention group, and PA combination with empagliflozin and 2 μmol/L Compound C (Sigma-Aldrich, 171261, St. Louis, MO, United States) intervention group.

### Reverse Transcription and Quantitative Real-Time PCR

Total RNA was isolated from liver tissues and cells by TRIzol Reagent (Invitrogen, 10296010, Carlsbad, CA, United States) and reverse transcribed into cDNA (Thermo Fisher Scientific, EP0442, Waltham, MA, United States). The primers were obtained from Tsing Ke Biological Technology (Beijing, China). RT-PCR was performed using a SYBR Green PCR reagent kit (Sangon Biotech, Shanghai, China) and the CFX Manager system (Bio-Rad, Berkeley, CA, United States). The relative expression levels of genes were normalised to β-actin. The primer sequences are listed in Supplementary Table 1.

### Western Blot

Tissue and cell proteins were separated by 10% SDS/PAGE gels and then transferred to nitrocellulose membranes (BioTrace™ NT, New York City, NY, United States). After blocking with 5% skim milk, the membrane was incubated with primary antibodies at 4°C overnight. The antibodies are listed as follows: total-AMPK (Abcam, ab80039, 1:2000, Cambridge, United Kingdom), phospho-AMPK at Thr172 (Abcam, ab23875, 1:2000, Cambridge, United Kingdom), total-CREB (Abcam, ab32515, 1:2000, Cambridge, United Kingdom), phospho-CREB at Thr133 (Abcam, ab32096, 1:2000, Cambridge, United Kingdom), total-GSK3β (Proteintech, 22104-1-AP, 1:1000, Wuhan, China), phospho-GSK3β at Ser389 (Proteintech, 14850-1-AP, 1:1000, Wuhan, China), PEPCK (Proteintech, 16754-1-AP, 1:1000, Wuhan, China), G6PASE (Novus, NBP1-80533, 1:1000, Littleton, CO, United States), and β-actin (Bioworld, BS6007M, 1:4000, Minneapolis, MN, United States). Then, HRP-conjugated secondary antibody (*Sungene Biotech*, LK 2001, *1*:*5000*, Tianjin, *China*) was incubated for 1 h at room temperature. Finally, the protein bands were developed by an ECL kit (Advansta, Menlo Park, CA, United States). The quantification of protein bands was analysed by ImageJ software.

### Periodic Acid–Schiff Staining

Periodic acid-schiff staining was used to detect glycogen deposits in the liver. Mouse liver tissues were fixed with 4% paraformaldehyde, mounted in paraffin, and sectioned at 4 μm. Paraffin sections were dewaxed in xylene and alcohol, and then rinsed three times in water. The paraffin sections were placed in periodic acid solution at room temperature and rinsed once with water. The sections were placed in Schiff reagent in the dark under dry conditions at room temperature and then rinsed with water. The sections were dyed with hematoxylin for 3 min (Leagene Biotechnology, Beijing, China). A neutral gum seal was used to dry the sections for subsequent microscopic observation. HL7702 cells were fixed with 4% paraformaldehyde and then subjected to PAS staining as described above.

### Glucose Production

The cells were collected and then disrupted by ultrasonication. The glucose production of each group was detected by a glucose content test kit (Solarbio, BC2505, Beijing, China). Standardised values for total protein content were measured from whole cell extracts (Lin et al., 2009).

### Enzyme Activities Test

The PEPCK and G6Pase activities were measured by detecting the rates of NADH decline at 340 nm by enzyme activity assay kit (Cablebridge Biotechnology, MS3500 and MS3501, Shanghai, China) according to the manufacturer’s protocols (Perez-Mendoza et al., 2014).

### Statistics Analysis

GraphPad Prism 5.0 software was used to analyse the statistical data, which are shown as the means ± SD. We used one-way ANOVA followed by Bonferroni’s test for comparisons among three or more groups. *P* < 0.05 was considered statistically significant.

## Results

### Effects of Empagliflozin on Blood Glucose and Body Weight

**FIGURE 1:**
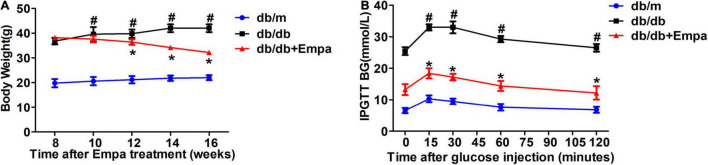
Effects of empagliflozin on blood glucose and body weight. **(A)** Body weight. **(B)** Intraperitoneal glucose tolerance test (IPGTT). *n* = 6 mice/group. *P < 0.05, db/db vs. db/db+Empa; ^#^*P <* 0.05, db/db vs. db/m.

Compared with db/db mice, the level of urinary glucose excretion was significantly increased after empagliflozin treatment (Li et al., 2021; Supplementary Figure 1A). The blood glucose level of Empa-treated mice was lower than that of db/db mice (Li et al., 2021; Supplementary Figure 1B). A decrease in the overall body weight of the Empa-treated group was observed compared with the db/db group (Figure 1A). In the IPGTT experiment, the blood glucose levels of the Empa-treated mice were significantly decreased at 0, 15, 30, 60, and 120 min relative to the db/db mice (Figure 1B). These results indicated that urinary glucose excretion, blood glucose levels and body weight could be significantly reduced by empagliflozin.

### Empagliflozin Could Partially Suppress Hepatic Gluconeogenesis and Promote Glycogen Synthesis by the AMPK/CREB/GSK3β Signalling Pathway

**FIGURE 2:**
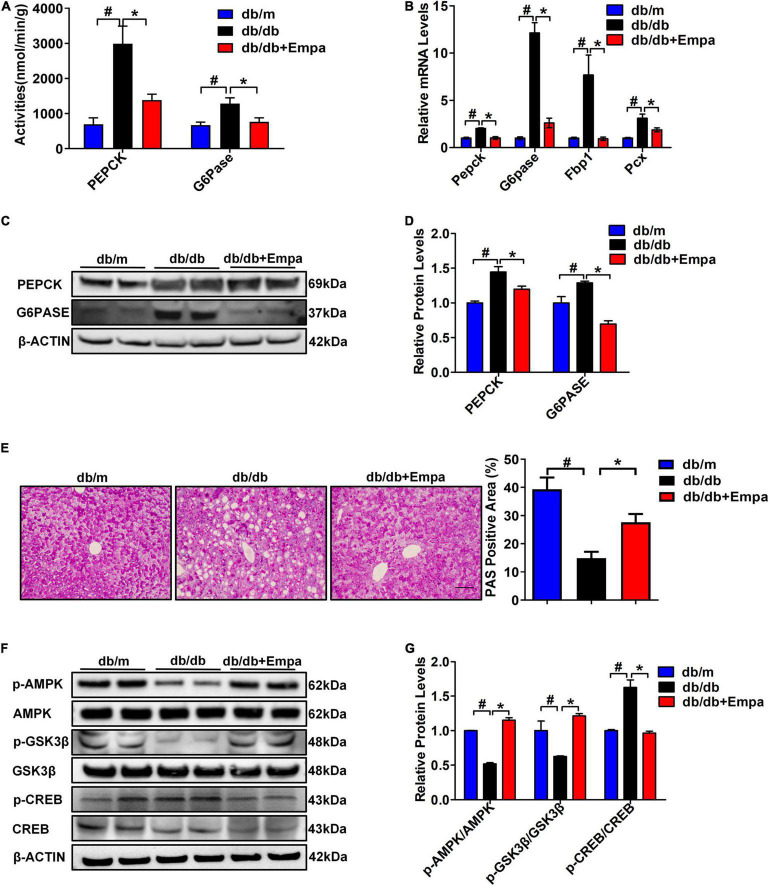
Empagliflozin partially suppressed hepatic gluconeogenesis and promoted glycogen synthesis. **(A)** Activities of PEPCK and G6Pase in hepatic tissues (*n* = 6 samples/group). **(B)** Relative mRNA expression levels of *Pepck*, *G6pase*, *Pcx*, and *Fbp1* in the liver were measured by RT-PCR (*n* = 6 samples/group). **(C)** Relative protein expression levels of PEPCK and G6PASE in the liver were determined by western blotting. **(D)** Protein quantitative statistics (*n* = 6 samples/group). **(E)** Hepatic PAS staining, scale bar = 100 μm (*n* = 6 samples/group). **(F)** Relative protein expression levels of p-AMPK, AMPK, p-GSK3β, GSK3β, p-CREB, and CREB in the liver were determined by western blotting. **(G)** Protein quantitative statistics (*n* = 6 samples/group). *P < 0.05, db/db vs. db/db+Empa; ^#^*P <* 0.05, db/db vs. db/m.

To further investigate the underlying molecular mechanisms of the hypoglycaemic effect of empagliflozin from the perspective of liver, we first measured the activities and expression levels of key enzymes involved in hepatic gluconeogenesis of hepatic tissues. Compared with db/db mice, the activities of PEPCK and G6Pase in Empa-treated mice were significantly decreased (Figure 2A). Then, the expression levels of key gluconeogenesis enzymes in the three groups of mice were detected. The relative mRNA expression levels of *Pepck*, *G6pase*, *Fbp1* (fructose-bisphosphatase 1) and *Pcx* (pyruvate carboxylase) in the Empa-treated mice were significantly lower than those in the db/db mice (Figure 2B). The relative protein expression levels of PEPCK and G6PASE were also consistent with the mRNA expression levels (Figures 2C,D). In addition, the PAS staining results showed that the reduced glycogen in db/db mice was restored after empagliflozin treatment (Figure 2E). Compared with that in db/m mice, the phosphorylation level of AMPK was significantly reduced in db/db mice. After empagliflozin treatment, the phosphorylation level of AMPK was significantly increased, although the phosphorylation level of CREB was significantly inhibited compared with that of the db/db mice. Furthermore, empagliflozin also increased the phosphorylation level of GSK3β (Figures 2F,G). These findings demonstrated that empagliflozin partially reduced hepatic gluconeogenesis and increased glycogen contents by the AMPK/CREB/GSK3β signalling pathway *in vivo*.

### Empagliflozin Could Inhibit Gluconeogenesis and Promote Glycogen Synthesis in HL7702 Cells

**FIGURE 3:**
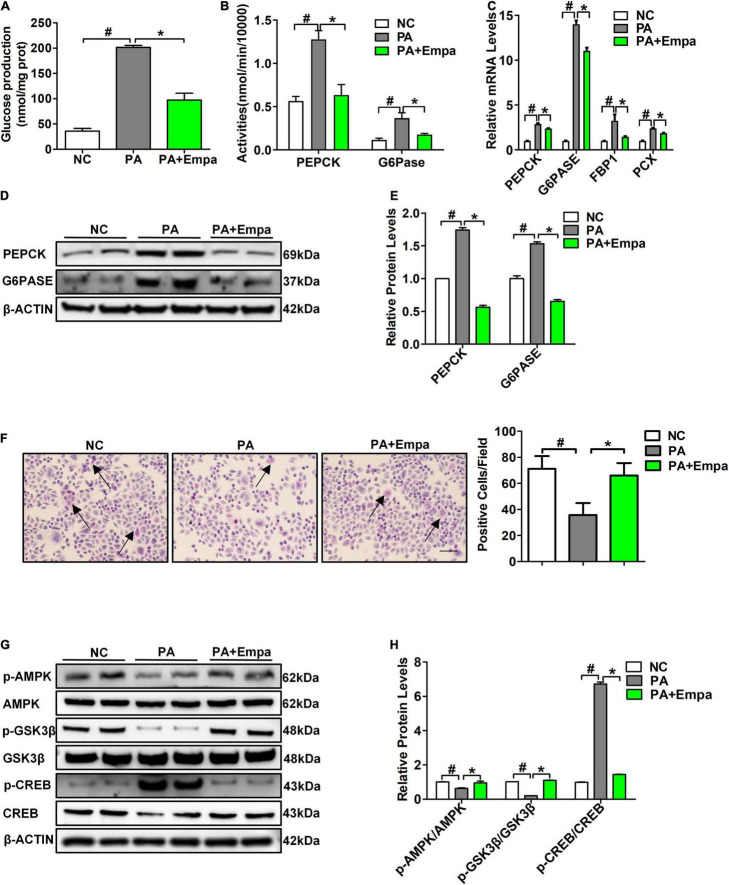
Empagliflozin inhibited gluconeogenesis and promoted glycogen synthesis in HL7702 cells. **(A)** Glucose production levels (*n* = 3 samples/group). **(B)** PEPCK and G6Pase activities (*n* = 3 samples/group). **(C)** Relative mRNA expression levels of *PEPCK, G6PASE, FBP1*, and *PCX* were measured by RT-PCR (*n* = 3 samples/group). **(D)** Relative protein expression levels of PEPCK and G6PASE were determined by western blotting (*n* = 3 samples/group). **(E)** Protein quantitative statistics (*n* = 3 samples/group). **(F)** PAS staining, scale bar = 100 μm (*n* = 6 samples/group). **(G)** Relative protein expression levels of p-AMPK, AMPK, p-GSK3β, GSK3β, p-CREB, and CREB were determined by western blotting. **(H)** Protein quantitative statistics (*n* = 3 samples/group). *P < 0.05, PA vs. PA+Empa; ^#^*P <* 0.05, NC vs. PA. Arrows indicate the areas of focus for PAS staining.

Palmitic acid treatment increased the relative mRNA expression levels of PEPCK and G6PASE in HL7702 cells. Different concentrations of 0 mmol/L, 0.25 mmol/L, 0.5 mmol/L, and 0.75 mmol/L PA were selected to treat HL7702 cells under the same conditions for 24 h. The highest mRNA expression levels of PEPCK and G6PASE were observed in the 0.75 mmol/L PA intervention group (Supplementary Figures 2A,B). Then, we selected 0.75 mmol/L PA for the following experiments. According to the results of the CCK-8 assay, 8 μmol/L empagliflozin was used to treat HL7702 cells (Supplementary Figure 2C). The glucose production content of the empagliflozin combination with PA group was significantly reduced compared with that of the PA treatment group (Figure 3A). The activities of PEPCK and G6Pase were also significantly decreased after empagliflozin combination with PA treatment (Figure 3B). Compared with the PA group, the relative mRNA and protein expression levels of PEPCK and G6PASE were obviously decreased in the empagliflozin combination with PA group (Figures 3C–E). PA reduced the GSK3β phosphorylation level and the glycogen content. The PAS staining results showed that the glycogen content of the empagliflozin combination with PA group was significantly increased compared with that of the PA treatment group (Figure 3F). Then we measured the protein expression levels of AMPK/CREB/GSK3β signalling pathway-related molecules, which are involved in the regulation of gluconeogenesis and glycogen synthesis. PA inhibited AMPK phosphorylation, while empagliflozin obviously activated the AMPK signalling pathway. The phosphorylation level of CREB was reduced after empagliflozin treatment. The phosphorylation level of GSK3β was increased in the Empa-treated group (Figures 3G,H). These results indicated that empagliflozin could rectify the enhanced gluconeogenesis and prevent reduced glycogen synthesis *in vitro*.

### 5-Aminoimidazole-4-carboxamide1-b-D-ribofuranoside and Compound C Could Regulate the AMPK Signalling Pathway in the Presence of Empagliflozin *in vitro*

**FIGURE 4:**
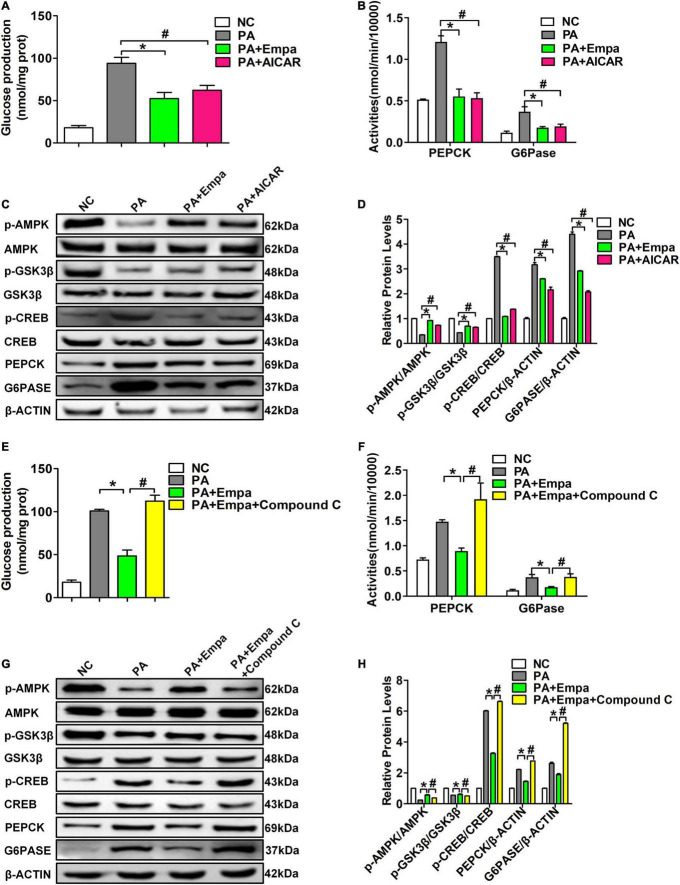
5-Aminoimidazole-4-carboxamide1-b-D-ribofuranoside (AIACR) and compound C could regulate the AMPK signalling pathway under empagliflozin treatment *in vitro*. HL7702 cells were treated with the AMPK agonist AICAR and empagliflozin and PA. **(A)** Glucose production levels (*n* = 3 samples/group). **(B)** PEPCK and G6Pase activities (*n* = 3 samples/group). **(C)** Relative protein expression levels of p-AMPK, AMPK, p-GSK3β, GSK3β, p-CREB, CREB, PEPCK, and G6PASE were determined by western blotting. **(D)** Protein quantitative statistics (*n* = 3 samples/group). *P < 0.05, PA vs. PA+Empa; ^#^*P <* 0.05, PA vs. PA+AIACR. HL7702 cells were treated with the AMPK inhibitor compound C and empagliflozin and PA. **(E)** Glucose production levels (*n* = 3 samples/group). **(F)** PEPCK and G6Pase activities (*n* = 3 samples/group). **(G)** Relative protein expression levels of p-AMPK, AMPK, p-GSK3β, GSK3β, p-CREB, CREB, PEPCK, and G6PASE were determined by western blotting. **(H)** Protein quantitative statistics (*n* = 3 samples/group). *P < 0.05, PA vs. PA+Empa; ^#^*P <* 0.05, PA+Empa vs. PA+Empa+Compound C.

To further verify the role of empagliflozin in activating the AMPK signalling pathway, AIACR, an activator of AMPK, was used to stimulate HL7702 cells in the empagliflozin and PA treatment groups. We found that both empagliflozin and AIACR could significantly reduce glucose production in HL7702 cells after PA treatment (Figure 4A). The activities of PEPCK and G6Pase in HL7702 cells were also consistent with the production of glucose (Figure 4B). Compared with the PA-treated group, the phosphorylation levels of AMPK were also obviously increased after empagliflozin and AIACR treatments. The phosphorylation levels of CREB were simultaneously reduced in both the Empa- and AIACR-treated groups. Both empagliflozin and AIACR could also increase the phosphorylation level of GSK3β. The protein expression levels of PEPCK and G6PASE were also decreased in the Empa- and AIACR-treated groups, which directly indicated the inhibition of gluconeogenesis (Figures 4C,D).

Compound C has been demonstrated to be a highly selective inhibitor of the AMPK signalling pathway. Compound C prevented the effect of empagliflozin in inhibiting the activities of PEPCK and G6Pase and the production of glucose in PA-treated HL7702 cells (Figures 4E,F). Compound C also abolished AMPK activation by empagliflozin in PA-treated HL7702 cells. The phosphorylation level of CREB was significantly increased by compound C. Compound C also decreased the phosphorylation level of GSK3β and increased the expression levels of PEPCK and G6Pase (Figures 4G,H). These results suggested that empagliflozin downregulated hepatic gluconeogenesis and upregulated glycogen synthesis by the AMPK/CREB/GSK3β signalling pathway *in vitro*.

## Discussion

The liver can effectively maintain the homeostasis of energy metabolism, especially in terms of glucose and lipids. When hungry or fasting, the liver can regulate the blood glucose level by glycogenolysis or gluconeogenesis *in vivo* (Tsintzas et al., 2013). The increase in endogenous glucose production and the decrease in hepatic glycogen storage contributed to metabolic abnormalities in T2DM (Biddinger and Kahn, 2006). Therefore, inhibition of hepatic glucose production may have a positive effect on the treatment of T2DM (Wu et al., 2005). Increasing hepatic glycogen synthesis and storage may also be beneficial for T2DM treatment. In this study, we demonstrated that empagliflozin could reduce hepatic gluconeogenesis and increase hepatic glycogen synthesis *in vivo* and *in vitro*, thus demonstrating the unique pharmacological effect of empagliflozin in addition to its role in hypoglycemia.

Glucose metabolism is a very complex process that is regulated by many mechanisms. As shown in Figure 1 and Supplementary Figure 1, we first selected a T2DM mouse model to investigate the effects of empagliflozin on glucose metabolism *in vivo*. Empagliflozin significantly reduced urinary glucose excretion and blood glucose levels, which suggested that empagliflozin had an obvious regulatory role in glucose metabolism (Figure 1 and Supplementary Figure 1). Next, we further explored the underlying mechanism by which empagliflozin regulates glucose metabolism. Most glucose utilisation and production are derived from hepatic gluconeogenesis *in vivo*. Hepatic gluconeogenesis is mainly regulated by some key enzymes, including PEPCK, G6Pase, PCX, and FBP1. As the rate-limiting enzymes of hepatic gluconeogenesis, the activities and expression levels of PEPCK and G6Pase were all significantly decreased after empagliflozin treatment, which suggested that empagliflozin influenced hepatic gluconeogenesis. Interestingly, we also found that empagliflozin could promote glycogen synthesis (Figures 2A–E). What are the molecular mechanisms by which empagliflozin regulates gluconeogenesis and glycogen synthesis? Previous studies have demonstrated that SGLT2 inhibitors can promote AMPK phosphorylation (Zhou et al., 2018; Chiang et al., 2020). We next tested the relative expression levels of AMPK/CREB/GSK3β signalling pathway-related molecules that are involved in the regulation of gluconeogenesis and glycogen synthesis. The relative expression levels of p-AMPK/AMPK, p-GSK3β/GSK3β, and p-CREB/CREB were all significantly changed (Figures 2F,G). Previous studies have demonstrated that activation of the AMPK signalling pathway inhibits the expression of TORC2, thus preventing it from binding with CREB to reduce CREB transcription levels and inhibit gluconeogenesis (Koo et al., 2005). Interestingly, activated AMPK could also induce an increase in the phosphorylation level of GSK3β, which results in an increase in the glycogen synthesis level (Horike et al., 2008). The above results preliminarily suggested that empagliflozin could induce the downregulation of PEPCK and G6Pase activities to inhibit hepatic gluconeogenesis *via* the AMPK/CREB signalling pathway and promote glycogen synthesis by activating the AMPK/GSK3β signalling pathway *in vivo*.

To further investigate whether empagliflozin can act on hepatocytes, we next selected PA in combination with empagliflozin to stimulate the human liver cell line HL7702 *in vitro.* As shown in Figure 3, empagliflozin significantly negatively regulated glucose production and the activities and expression levels of PEPCK and G6Pase in HL7702 cells. The changes in glycogen content were also consistent with those *in vivo*. The relative expression levels of p-AMPK/AMPK, p-GSK3β/GSK3β, and p-CREB/CREB were all significantly changed *in vitro* (Figure 3). The above results demonstrated that empagliflozin could rectify enhanced gluconeogenesis and prevent reduced glycogen synthesis in hepatocytes. Next, we selected the specific AMPK agonist AICAR and the inhibitor compound C to further verify the effects of the AMPK signalling pathway on hepatic gluconeogenesis and glycogen synthesis. As shown in Figure 4, the results of the AIACR intervention were consistent with those of empagliflozin treatment in HL7702 cells, and the effects of empagliflozin treatment could be abolished by compound C, which further demonstrated that empagliflozin could regulate gluconeogenesis and glycogen synthesis by the AMPK signalling pathway (Figure 4).

In the process of maintaining glucose homeostasis, insulin and glucagon are highly effective regulatory factors for blood glucose, which are secreted by islet cells (Campbell and Newgard, 2021). Recently, clinical studies have reported that empagliflozin can decrease endogenous glycerol-gluconeogenesis in T2DM patients with obesity, which was partially consistent with our experimental results (Neeland et al., 2020). In this study, we systematically demonstrated that empagliflozin can inhibit hepatic gluconeogenesis and promote glycogen synthesis by the AMPK/CREB/GSK3β signalling pathway *in vivo* and *in vitro*. However, there were still some constraints in this study. First, although we have demonstrated that empagliflozin inhibits hepatic gluconeogenesis and promotes glycogen synthesis by activating AMPK, whether AMPK is directly activated by empagliflozin is still worthy of further investigation. Second, the sequence of gluconeogenesis and glycogen synthesis that are regulated by empagliflozin is still unknown and will represent an interesting research focus in the future. Third, although we focused on tAMPK/CREB/GSK3β, empagliflozin might reduce hepatic gluconeogenesis and increase glycogen synthesis by different signalling pathways; thus, additional signalling pathways should be further studied.

## Conclusion

In addition to traditional renal and cardiovascular benefits, empagliflozin could partially inhibit hepatic gluconeogenesis and promote glycogen synthesis by the AMPK/CREB/GSK3β signalling pathway *in vivo* and *in vitro*. This study focused on the hepatic benefits of empagliflozin, which showed the pharmacological effects of empagliflozin more comprehensively. Our findings provide new insights into empagliflozin as a rising star of hypoglycaemic drugs for T2DM treatment and thus provide a foundation for further research focusing on glucose metabolism.

## Data Availability Statement

The original contributions presented in the study are included in the article/Supplementary Material, further inquiries can be directed to the corresponding author/s.

## Ethics Statement

The animal study was reviewed and approved by the Experimental Animals Ethics Committee of Tianjin Medical University, Chu Hsien-I Memorial Hospital and Tianjin Institute of Endocrinology.

## Author Contributions

XY, ZM, and TF performed the experiment, analyzed the data, and wrote the manuscript. XhL, YC, LX, XyL, and XL assisted in the experiments. MX and TL helped to analyze the data. LC and BS designed the experiment, evaluated and reviewed the manuscript structure, ideas, and science. All authors read and approved the final manuscript.

## Conflict of Interest

The authors declare that the research was conducted in the absence of any commercial or financial relationships that could be construed as a potential conflict of interest.

## Publisher’s Note

All claims expressed in this article are solely those of the authors and do not necessarily represent those of their affiliated organizations, or those of the publisher, the editors and the reviewers. Any product that may be evaluated in this article, or claim that may be made by its manufacturer, is not guaranteed or endorsed by the publisher.

## Supplementary Material

Supplementary Figure 1 Effects of empagliflozin on urinary glucose excretion and fasting blood glucose. **(A)** Urine glucose excretion. **(B)** Fasting blood glucose (*n* = 6 mice/group). **P* < 0.05, db/db vs. db/db+Empa; ^#^*P* < 0.05, db/db vs. db/m.

Supplementary Figure 2 Effects of different concentrations of empagliflozin and PA in HL7702 cells. **(A,B)** Relative mRNA expression levels of *PEPCK* and *G6PASE* in HL7702 cells treated with different concentrations of PA for 24 h (*n* = 3 samples/group). **(C)** The viabilities of HL7702 cells treated with different concentrations of empagliflozin was detected by CCK-8 assay (*n* = 3 samples/group). **P <* 0.05, 0.75 mmol/L PA vs. NC.

## Abbreviations

AMPK: AMP-activated protein kinase
AIACR: 5-Aminoimidazole-4-carboxamide1-b-D-ribofuranoside
CREB: cAMP-responsive element binding protein
FBP1: fructose-bisphosphatase 1
G6Pase: glucose-6-phosphatase
GS: glycogen synthase
GSK3β: glycogen synthase kinase-3β
IPGTT: intraperitoneal glucose tolerance tests
PA: palmitic acid
PAS: periodic acid-schiff
PEPCK: phosphoenolpyruvate carboxykinase
PCX: pyruvate carboxylase
SGLT2: sodium-dependent glucose transporters 2
T2DM: type 2 diabetes
TORC2: CREB-regulated transcription coactivator 2.
