# Supplementary figures and images for: Empagliflozin Inhibits Hepatic Gluconeogenesis and Increases Glycogen Synthesis by AMPK/CREB/GSK3β Signalling Pathway
Source: Front Physiol. 2022 Mar 1;13:817542. doi: 10.3389/fphys.2022.817542 (PMC8921641; doi:10.3389/fphys.2022.817542)

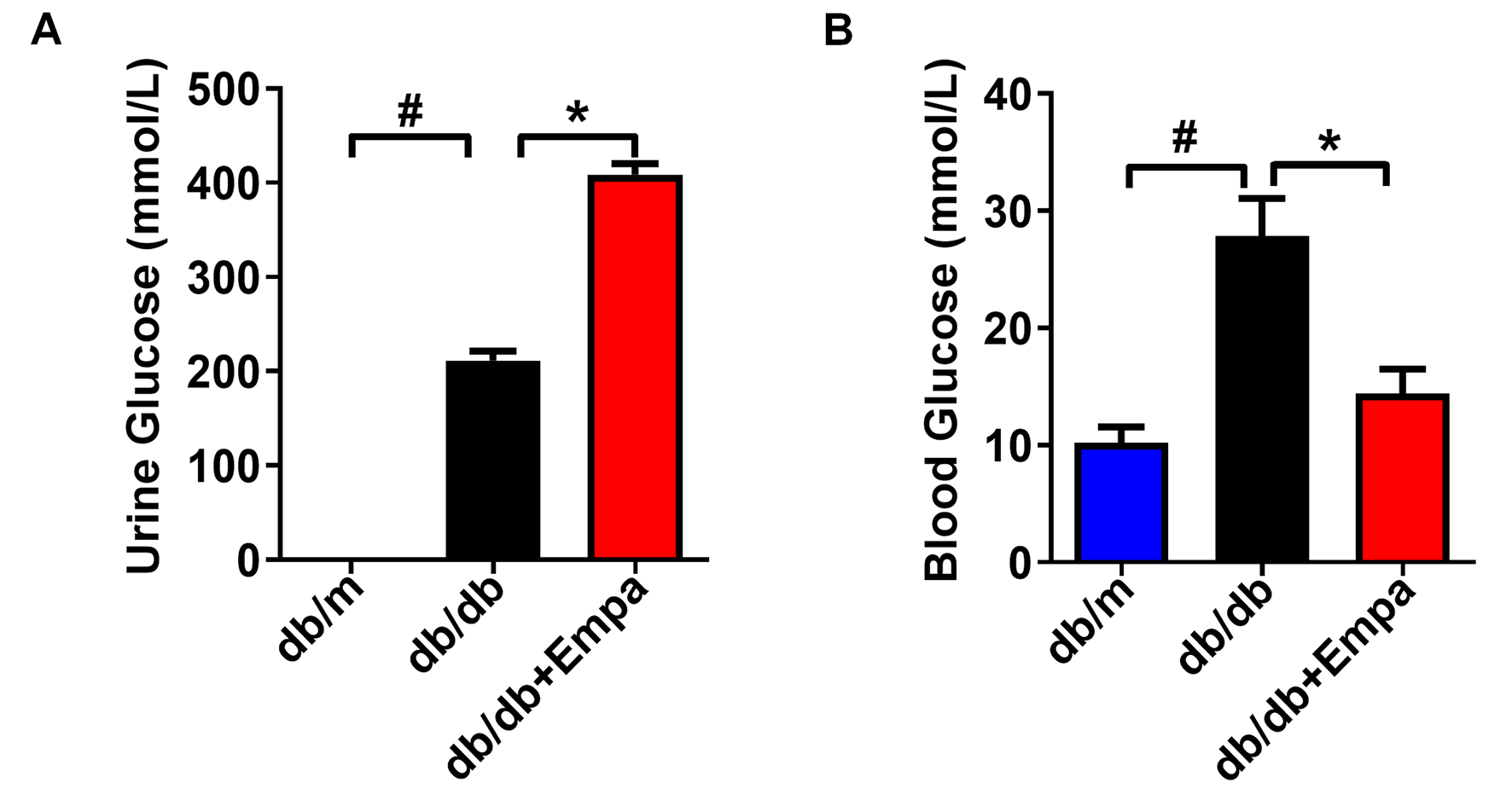

Supplement: Supplementary Figure 1 — Effects of empagliflozin on urinary glucose excretion and fasting blood glucose. (A) Urine glucose excretion. (B) Fasting blood glucose (n = 6 mice/group). *P < 0.05, db/db vs. db/db+Empa; #P < 0.05, db/db vs. db/m. [file Image_1.TIF]

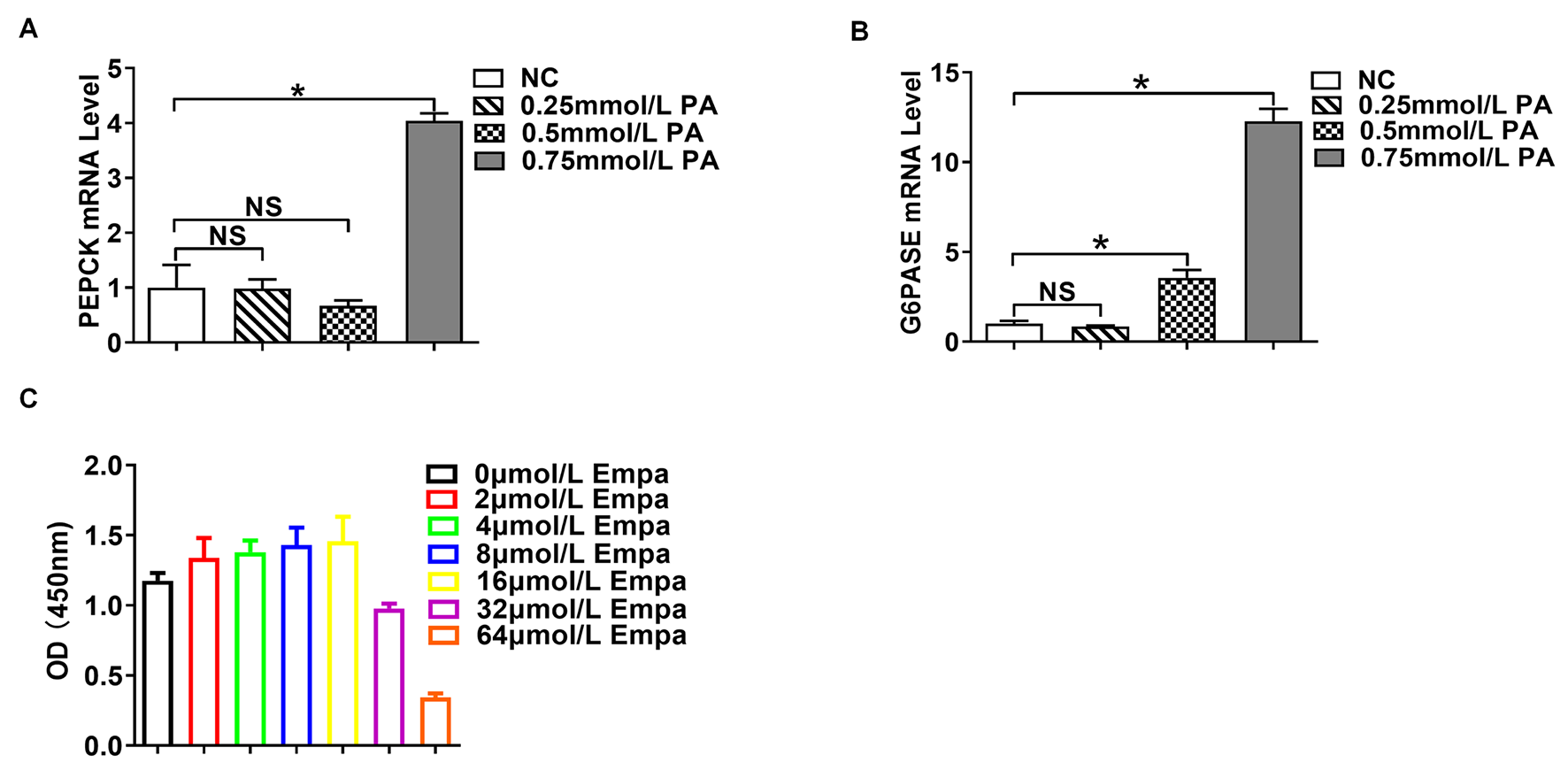

Supplement: Supplementary Figure 2 — Effects of different concentrations of empagliflozin and PA in HL7702 cells. (A,B) Relative mRNA expression levels of PEPCK and G6PASE in HL7702 cells treated with different concentrations of PA for 24 h (n = 3 samples/group). (C) The viabilities of HL7702 cells treated with different concentrations of empagliflozin was detected by CCK-8 assay (n = 3 samples/group). *P < 0.05, 0.75 mmol/L PA vs. NC. [file Image_2.TIF]
